# Sphingosine 1-Phosphate Signaling and Metabolism in Chemoprevention and Chemoresistance in Colon Cancer

**DOI:** 10.3390/molecules25102436

**Published:** 2020-05-23

**Authors:** Petra Grbčić, Mirela Sedić

**Affiliations:** Department of Biotechnology, University of Rijeka, Radmile Matejčić 2, 51000 Rijeka, Croatia; petra.grbcic@biotech.uniri.hr

**Keywords:** colon cancer, chemoresistance, sphingosine 1-phosphate, sphingolipid metabolism, sphingosine kinases

## Abstract

Colorectal carcinoma (CRC) is the leading cause of cancer-related deaths worldwide. Despite advances in prevention and treatment modalities for CRC, rapidly developing resistance to chemotherapy limits its effectiveness. For that reason, it is important to better understand the mechanisms that undergird the process of chemoresistance to enable design of novel anticancer agents specifically targeting malignant properties of cancer cells. Over recent decades, bioactive sphingolipid species have come under the spotlight for their recognized role in cancer development and progression, and the evidence has surfaced to support their role as regulators of anti-cancer drug resistance. Colon cancer is characterized by a shift in sphingolipid balance that favors the production and accumulation of oncogenic species such as sphingosine 1-phosphate (S1P). S1P is known to govern the processes that facilitate cancer cell growth and progression including proliferation, survival, migration, invasion and inflammation. In this review paper, we will give a comprehensive overview of current literature findings on the molecular mechanisms by which S1P turnover, transport and signaling via receptor-dependent and independent pathways shape colon cancer cell behavior and influence treatment outcome in colon cancer. Combining available modulators of S1P metabolism and signaling with standard chemotherapy drugs could provide a rational approach to achieve enhanced therapeutic response, diminish chemoresistance development and improve the survival outcome in CRC patients.

## 1. Introduction into Sphingosine 1-Phosphate Signaling

**Figure 1:**
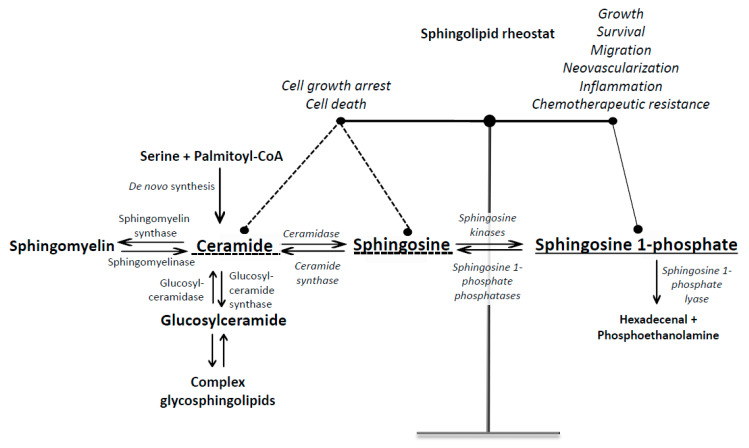
Simplified scheme of sphingolipid metabolism and turnover. Ceramide can be produced either de novo from serine and palmitoyl-CoA or by hydrolysis of complex sphingolipids (sphingomyelin and glycosphingolipids). S1P is produced by hydrolysis of ceramide to sphingosine by ceramidases followed by the phosphorylation of sphingosine by sphingosine kinases. S1P can be either dephosphorylated back to sphingosine by S1P phosphatase or irreversibly cleaved by S1P lyase into phosphoethanolamine and hexadecenal. Ceramide, sphingosine and S1P have opposing functions, and their intracellular balance is a critical regulator of cell fate.

Sphingosine 1-phosphate (S1P), a bioactive lipid mediator, is found in various cancerous tissues as well as in tumor microenvironment (TME). S1P governs the processes that drive cancer cell growth and progression including proliferation, survival, migration, invasion, angiogenesis, lymphangiogenesis and inflammation [1]. The dynamic balance between pro-survival S1P and pro-apoptotic sphingolipid species including ceramide and sphingosine, the so-called sphingolipid rheostat, is deemed critical for determining cell fate (Figure 1). Thus, the agents that shift the balance between ceramide-sphingosine-S1P can steer the cells towards either apoptosis or survival depending on the relative position of the rheostat [2]. The intracellular concentration of S1P is low and tightly controlled by the balance between its production catalyzed by sphingosine kinases, dephosphorylation back to sphingosine by S1P phosphatases and lipid phosphate phosphatases (LPPs), and its catabolism catalyzed by endoplasmic reticulum enzyme S1P lyase. There are two isotypes of sphingosine kinases that differ in their tissue distribution, subcellular localization and conformation and dimerization properties. Sphingosine kinase 1 (SphK1) is mainly localized in the cytosol and generates S1P that is transported outside the cell to the extracellular space including the tumor microenvironment [3]. Oppositely, sphingosine kinase 2 (SphK2) is found predominantly in the nucleus, mitochondrion and endoplasmic reticulum and produces S1P that binds primarily to intracellular targets [4]. Both enzymes can be exported from the cells. Thus, SphK1 can be secreted by endothelial cells [5], whereas SphK2 is cleaved by caspase-1 in the cells undergoing apoptosis and its truncated, enzymatically active fragment released into the extracellular space [6]. Extracellular sphingosine kinases as well as secretion of S1P from the cells via autocrine or paracrine signaling contribute to the availability of circulating S1P. S1P in the circulation is bound to albumin, low-density lipoproteins and high-density lipoproteins, and can be also carried by erythrocytes. Extracellular S1P is dephosphorylated into sphingosine mainly by the ecto-activities of LPPs which facilitate cellular entry of sphingosine produced from S1P outside the cells [7]. Once inside the cells, sphingosine is phosphorylated back to S1P which triggers intracellular signaling events.

Mammalian LPPs consist of three isoforms: LPP1, LPP2, and LPP3. Previous studies have demonstrated low levels of expression of LPP1 and LPP3 in different cancer cells and tumors, whereas increased expression of LPP2 has been correlated with cell transformation and accelerated entry into S-phase of the cell cycle, which suggests that LPP2 has an opposite effect on cancer cell growth in comparison with LPP1 and LPP3 [8]. Raising the expression of LPP1 in breast and thyroid cancer cells attenuates tumor growth and suppresses lung metastasis in mice, which could be attributed to inhibition of signaling downstream of G-protein-coupled receptors [9]. Similarly, ovarian carcinoma cells with forced expression of human LPP3 exerted reduced tumor growth in nude mice [10]. Since the ecto-activity of LPP1 and LPP3 can contribute to the degradation of S1P [7] whose increased production and secretion is associated with the development of chemoresistance and stimulation of angiogenesis to support tumor growth, LPP1 and LPP3 could play important roles in these processes by controlling the levels of extracellular S1P.

Different stimuli including growth factors and cytokines such as epidermal growth factor (EGF), platelet derived growth factor (PDGF), vascular endothelial growth factor (VEGF), insulin-like growth factor- 1 (IGF-1), tumor necrosis factor α (TNFα) and interleukin-1β (IL-1β), hormones (e.g., oestrogen, estradiol) and hypoxia can activate SphK1 which then translocates from cytosol to the plasma membrane and phosphorylates sphingosine to produce S1P. Produced S1P is exported from cancer cells by specific transporter proteins that reside in the plasma membrane into extracellular space where it binds to and activates specific G protein–coupled receptors located on the plasma membrane in an autocrine or paracrine manner (known as inside-out signaling) to promote cancer cell proliferation, migration, invasion and survival.

**Figure 2:**
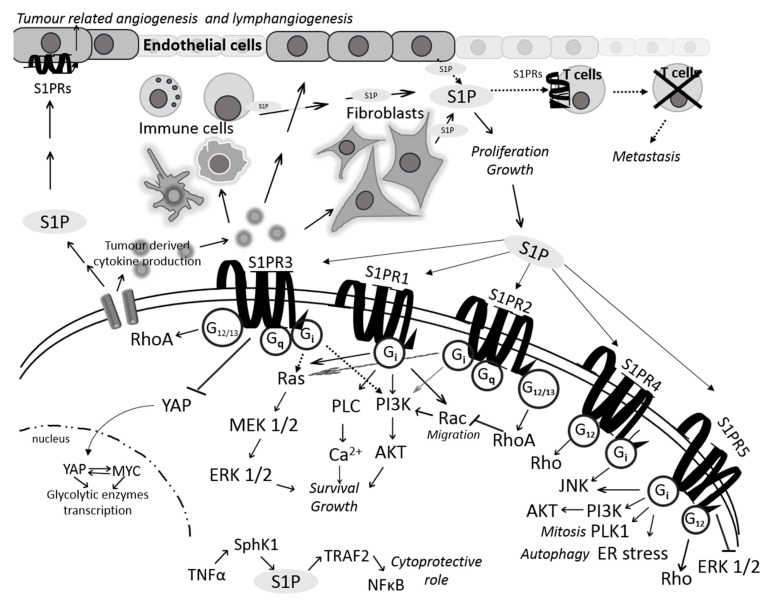
S1P-mediated interactions between cancer cells and the components of tumor microenvironment (TME). Tumor cells enable cytokine production that, when released in TME, stimulate endothelial cells, fibroblasts or immune cells to produce and secrete S1P. In this way, cancer cell ensures its survival and proliferation. On the other hand, S1P secreted from cancer cells can influence endothelial cells to promote angiogenesis and lymphangiogenesis but also suppress the activity of cytotoxic T-cells. Secreted S1P also binds to five different plasma membrane G protein-coupled receptors (S1PR1–5). Activated receptors, through the interaction with various associated G proteins, can regulate survival, proliferation, angiogenesis and migration by inducing several oncogenic signaling pathways such as RAS/MEK/ERK and PI3K/AKT. In comparison with S1P receptors 1, 3, 4 and 5, S1PR2 is known to bear dual role via either inhibition or activation of RAS/ERK and PI3K/AKT signaling due to its ability to couple to different G proteins.

S1P mediates interaction between cancer cells and cellular components of the tumor microenvironment via tumor-derived cytokine production that induces stromal cells (such as fibroblasts, immune cells, and endothelial cells) in the tumor microenvironment to produce and secrete S1P, which in turn promotes cancer cell proliferation and survival [3]. S1P secreted from cancer cells activates S1P receptors on endothelial cells to promote tumor-related angiogenesis and lymphangiogenesis. S1P secreted from vascular endothelial cells (EC) contributes to circulating levels of S1P and can affect endothelial barrier function by activating S1P receptors on endothelial cells [11]. In addition, circulating S1P secreted by endothelial cells can promote cancer metastasis by suppressing the activity of cytotoxic T-cells through binding to S1P receptors on the surface of immune cells (Figure 2) [12]. Both systemic and tumor S1P are important regulators of local tumor growth; however, it is systemic S1P generated by sphingosine kinase 1 rather than tumor-derived S1P that controls tumor metastasis, and inhibition of systemic S1P signaling using anti-S1P monoclonal antibody Sphingomab is able to suppress lung colonization and metastasis of urothelial carcinoma cells [13]. Thus, circulating S1P acts as a mediator of host–cancer cell communication on the road to cancer metastasis.

Secreted S1P transduces signals by binding to five different plasma membrane G protein-coupled receptors (S1P_1–5_), whose expression is tissue-specific. These receptors couple to different G proteins that control various downstream signal transduction pathways including Rho family, JNK, AKT/ERK, PLC/Ca^2+^ and adenylate cyclase to promote survival, proliferation, angiogenesis and migration. Depending on the cell type, the level of S1P receptor expression and the presence of associated G protein, S1P signaling could produce different cellular outcomes. S1PR1 is coupled exclusively to G_i_ protein whose activation results in activation of Ras/MEK/ERK and PI-3-kinase/AKT pathways to promote mitogenic and pro-survival signaling, and also in activation of Rho family small GTPase Rac, which regulates cell migration and formation of lamellipodia [14]. S1PR1 could also activate phospholipase C (PLC) to induce Ca^2+^ mobilization. S1PR2 has opposing roles in cancer cells which seems to depend on specific molecular and cellular context given that S1PR2 can couple to multiple Gα proteins, such as G_i_, G_12/13_ and G_q_, and can thus induce different signaling events. Coupling of S1PR2 to G_12/13_ results in activation of RhoA and downstream inhibition of Rac and AKT to inhibit cell migration and cell proliferation, respectively. Oppositely, S1PR2 can also activate Ras/ERK and PI-3-kinase pathways through G_i_ activation to promote proliferation and suppress apoptosis. S1PR3 also couples to different G proteins including Gi, G_12/13_ and G_q_. Similar to S1PR1, S1PR3 promotes migration through the Rac pathway via G_i_. In addition, S1PR3 triggers signaling via Ras/ERK and PI-3-kinase via G_i_ to promote mitogenic/pro-survival effects of S1P. S1P could also regulate cancer cell metabolism via S1PR3 by accelerating aerobic glycolysis (Figure 2) [15].

The roles of S1PR4 and S1PR5 in cancer have not been completely elucidated. These receptors are known to couple to Gi and G_12/13_ in response to S1P. S1PR4 is predominantly found in the lymphoid system, which indicates its important role in immune response (Figure 2) [16]. Recent study has provided evidence to support the role of S1PR4 as a potential regulator of antitumor immunity. S1PR4 deficiency leads to a decrease in breast cancer metastasis to lung and suppression of tumor growth accompanied by reduction in suppressive immune cells and increased number of tumoricidal lymphocytes, namely cytotoxic CD8+ T-cells [17]. Importantly, S1PR4-deficient tumors showed significantly increased response to treatment with doxorubicin in comparison with wild-type tumors, which was strictly dependent upon CD8+ T-cells. Depletion of S1PR4 potentiated the effect of anti-PD-1 immunotherapy in a synergistic manner resulting in reduced tumor growth and increased survival rate of tumor-bearing S1PR4-/- compared to WT mice [17]. Results from this study lend support to the conclusion that targeting S1PR4 could prove as an efficient strategy to restore anticancer immunity and improve cancer cell response to immunotherapy.

S1PR5 has been demonstrated to support cancer cell growth and survival. For example, Sphk1-derived S1P promotes mitosis in human cervical carcinoma HeLa cells by binding to and activating S1PR5 on the extracellular side resulting in the downstream activation of intracellular phosphatidylinositol 3-kinase (PI3K)-AKT signaling pathway [18]. The same study revealed the mitotic kinase Polo-like kinase 1 (PLK1) to be an important mediator of S1P-S1PR5-induced mitosis in HeLa cells (Figure 2). Study in prostate cancer cells corroborated pro-survival role of S1PR5 by demonstrating that exogenous S1P-induced autophagy under serum-deprived conditions was associated with S1PR5 activation [19]. Further study revealed that S1P-S1PR5 signaling-induced autophagy in these cells resulted from the induction of endoplasmic reticulum stress [20].

Besides its ability to exert biological effects in receptor-dependent manner, intracellular S1P can also regulate cancer cell signaling independently of its cell surface G-protein-coupled receptors. Cytosolic S1P produced by SphK1 has been shown to have different functions in comparison with S1P formed by SphK2 at the endoplasmic reticulum and/or other membranes [21]. For example, the study in human cervical carcinoma cell line HeLa has demonstrated that intracellular S1P produced by TNF-α-activated SphK1 specifically binds to the ubiquitin ligase TNF receptor-associated factor 2 (TRAF2) in the cytoplasm and triggers downstream molecular events ultimately resulting in the activation of NF-κB signaling, which could, at least partially, account for cytoprotective function of S1P (Figure 2) [22]. Additional study has shown that intracellular S1P directly binds to the transcription factor peroxisome proliferator-activated receptor (PPAR)γ in human endothelial cells and induces the association of PPARγ with peroxisome proliferator-activated receptor-γ coactivator 1 (PGC1β) in endothelial cells [23]. Importantly, the same study demonstrated that the interaction between S1P and (PPAR)γ was important for neovascularization, which suggests that S1P-PPARγ axis could be a novel target to impair in vivo angiogenesis and suppress tumor progression.

S1P produced by SphK2 in the mitochondria and nucleus has different intracellular targets. SphK2-produced S1P in the nucleus is involved in the epigenetic regulation of gene expression via binding to and inhibiting histone deacetylases HDAC1 and HDAC2, which enhances acetylation of histones leading to induction of *p21* and c-*fos* gene transcription in MCF-7 human breast cancer cells [24]. S1P produced in the inner mitochondrial membrane by SphK2 binds to prohibitin 2 (PHB2), protein that plays a crucial role in regulating mitochondrial assembly and function [25]. The interaction between mitochondrial S1P and PHB2 is central to proper assembly of cytochrome-c oxidase and mitochondrial respiration and thus plays an important role in maintaining mitochondrial function.

## 2. Sphingosine 1-Phosphate Transporters in Colon Cancer

As previously mentioned, sphingosine 1-phosphate generated by SphK1 inside cancer cells is exported to the extracellular matrix, where it signals via G protein-coupled S1P receptors on the cell membrane. Due to polar head group attached to its backbone, S1P has a hard time passing through the membrane and thus requires transporter proteins to traverse the plasma membrane. S1P is actively transported out of the cell by non-specific ABC-binding cassette transporters and through S1P-specific transporters including MFSD2B, S1P transporter from erythrocytes and platelets [26] and spinster homologue 2 (SPNS2), the latter being specifically active in endothelial cells and having a central role in the migration of lymphocytes from the thymus and secondary lymphoid organs into the blood [27].

ABC transporters have been considered as important molecular factors in tumorigenesis and development of chemoresistance in many cancer types [28,29]. Among these, ATP-binding cassette sub-family C member 1 (ABCC1), ATP-binding cassette sub-family G member 2 (ABCG2) and ATP-Binding Cassette Subfamily A Member 1 (ABCA1) play an important role in actively transporting S1P out of the cell. Growing evidence confirms that an intricate interplay exists between S1P metabolism and the regulation of the activity of specific ABC transporters. For example, in cerebral endothelial cells, SphK1 activity positively regulates the expression of P-glycoprotein (encoded by the *ABCB1* (*MDR1*) gene), and S1P induces P-gp transport activity via S1P receptors S1PR1 and S1PR3 located on the endothelial cell surface [30].

Several different studies lead unambiguously to the conclusion that ABC transporters involved in the export of S1P from the cells play a central role in the development of chemoresistance in colon cancer. For example, the importance of ABCC1 in colon cancer chemoresistance was demonstrated in the study showing that expression of ABCC1 at the gene and protein level was increased in multidrug resistant colon cancer cell lines in comparison with their sensitive counterpart [31]. The same study revealed that down-regulation of ABCC1 expression by miR-133b increases the sensitivity of colon cancer cells to anti-tumor drugs 5-fluorouracil and vincristine. Similarly, human colon cancer cells with acquired resistance to irinotecan have an increased expression of ABCG2 in comparison with sensitive cells [32]. Pharmacological inhibition of ABCG2 transporter by Ko143 enhanced the sensitivity of resistant cells to irinotecan. The involvement of ABCG2 in irinotecan resistance was corroborated in clinical samples from colon cancer patients demonstrating increased levels of the ABCG2 mRNA in hepatic metastases after irinotecan-based chemotherapy in comparison with irinotecan-naive metastases. Furthermore, ABCG2 expression was shown to be an important prognostic factor for the progression and metastasis of CRC and more abundant in CRC patients with positive lymph nodes [30]. Similarly, ABCG2 and ABCB1 were reported to be up-regulated in the response to combined 5-FU and irinotecan treatment in SW480 colorectal carcinoma cell line concomitant with the activation of Wnt signaling [33].

Overexpression of ABCA1 is linked with advanced stages of colorectal carcinoma and was shown to be important for stabilization of caveolin-1 to promote colon cancer cell migration and invasion towards cancer progression and metastasis [34]. This process could be associated with S1P production and secretion, since translocation of activated SphK1 from the cytosol to the plasma membrane domains enriched in caveolin-1 may promote export of S1P into the extracellular space and enable autocrine S1P signaling [35].

Another transporter, Spinster homologue 2 (Spns2) is closely linked with the regulation of S1P levels to augment cancer cell survival, growth, proliferation, metastasis and chemoresistance [36,37,38]. Little is known about the Spns2 regulation in colon cancer, but findings from the studies in other cancer types such as cervix adenocarcinoma underline the importance of Spns2-induced S1P secretion for promotion of cancer cell invasion in response to EGF signaling [39]. An additional clue for the involvement of Spns2 in the induction of metastasis was provided by the study in mice models of metastatic melanoma with Spns2 knockout that exerted low rate of metastasis formation in lung nodules accompanied by enhanced immune defence [12], which is in line with the role of S1P as a regulator of immune response, inflammation, angiogenesis and lymphomagenesis to promote cancer metastasis [40,41,42].

It has been documented that Spns2 mRNA and protein levels are highly expressed in colon cancer tissues in comparison with adjacent non-cancerous tissues and to correlate with tumor size, which points to the role of Spns2 in colon cancer progression [43,44]. Gu et al. [43] have reported that Spns2 induces proliferation, migration and invasion and suppresses apoptosis in human colon cancer cells by activating S1P/S1PR1/S1PR3 signaling axis and inducing AKT and ERK signaling pathways, which implicates that Spns2 could be considered as a promising therapeutic target in treating colon cancer. Overexpression of Spns2 is believed to initiate compensatory mechanism by which colon cancer cells restore S1P levels by upregulating SphK1 and SphK2 [45] and activate ERK and AKT signaling pathways that are by all means important for cell survival and cancer progression [43].

## 3. Sphingosine 1-Phosphate Receptors in Colon Cancer

Sphingosine 1-phosphate receptors have been receiving much attention due to their demonstrated role in the development of cancer, promotion of cancer cell growth and proliferation, and particularly in the development of chemoresistance. Because of high specificity and affinity for S1P, they are considered important for maintaining the sphingolipid signaling. Findings on the expression levels of S1P receptors and their functional roles in colon cancer pathogenesis are inconsistent across different studies. For example, Shida et al. [46] detected a variable expression of S1P receptors including S1PR1, S1PR2 and S1PR3 at the gene level in colorectal cancer without a specific pattern and significant alteration between cancer and normal tissues. On the contrary, findings from the study based on tissue microarray and immunohistochemical analyses revealed significantly higher expression of the S1PR1 protein in the colorectal cancer lesions in comparison with adjacent non-cancerous tissues [47]. The same study found that increased expression of S1PR1 was correlated with metachronous liver metastasis and poor overall survival in colorectal cancer patients and was recognized as an independent prognostic factor. Similarly, Uranbileg et al. [44] reported on the increased mRNA levels of S1PR2 and S1PR3 receptors in colon cancer tissues compared with the adjacent non-tumorous tissues. Growth-promoting role of S1PR2 was also shown in normal rat intestinal crypt cells, where down-regulation of S1PR2 abolished S1P-induced cell proliferation and migration by inhibiting S1P-mediated activation of ERK1/2 [48]. However, results from these studies contrast those by Petti et al. [49], who showed that lack of S1PR2 promotes tumor formation, growth and progression in mouse models of colorectal cancer. Given that the expression of S1PR2 was elevated in benign adenomas but markedly declined in carcinoma, these authors hypothesized that S1PR2 may have a tumor suppressor role in colon cancer. Further studies are warranted to ascertain the role of S1PR2 in regulating the behavior of colon cancer cells and determine its therapeutic significance.

The up-regulation of S1PR1 activity has a central role in sustaining persistent activation of transcription factors NF-κB and STAT3 in a feed-forward amplification loop that promotes chronic inflammation and colitis-associated cancer [40]. Up-regulation of SphK1 in acute colitis and colitis-associated cancer leads to an increased production of S1P. Secreted S1P binds to and activates S1PR1 leading to the activation of transcription factor STAT3, which in turn induces the expression of S1PR1. Intracellular S1P activates NF-κB and induces elevation of IL-6 that ultimately activates STAT3 transcription factor leading to the increased expression of S1PR1 and its constitutive activation. Up-regulation of S1PR1 activity results in the persistent activation of STAT3 and the resulting rise in the IL-6 expression.

**Figure 3:**
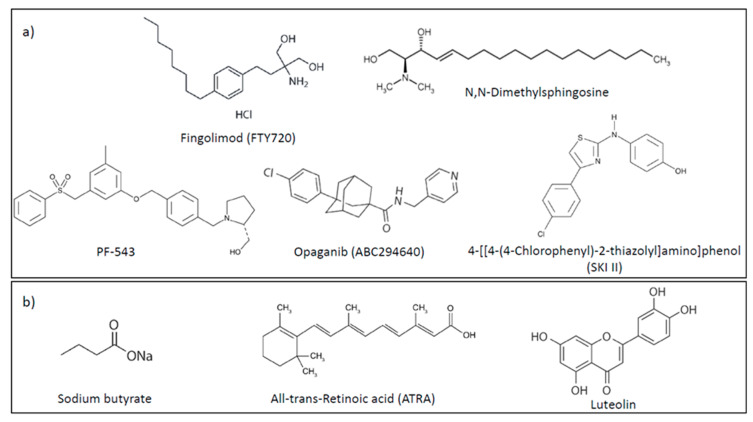
Chemical structures of compounds targeting S1P metabolism and signaling. (**a**) synthetic compounds; (**b**) natural compounds.

FTY720 (Fingolimod, Gilenya) (Figure 3) is an oral sphingosine analogue used in the treatment of relapsing multiple sclerosis patients. Immunosuppressive activity of FTY720 depends on its phosphorylation by sphingosine kinase 2 into FTY720-P that binds to S1P receptor 1 (S1PR1) in the lymphocytes’ membranes and induces internalization and degradation of S1PR1 leading to its loss from the plasma membrane and sustained down-regulation at the gene expression level [50]. As a consequence, the egress of lymphocytes from lymphoid organs into the circulation to the sites of inflammation is prevented. Importantly, FTY720 has been demonstrated to inhibit tumor growth and angiogenesis in vivo without toxic effects on normal cells [51]. In vitro studies have also revealed the ability of FTY720 to induce apoptosis and inhibit growth of different cancer cells by suppressing mitogenic and pro-survival signaling. Opposite to FTY720, its phosphorylated derivative FTY720-P exerts different effects in cancer cells. Thus, FTY720-P does not inhibit the growth of human breast cancer and colon cancer cell lines in the concentration range of 5–50 µM and exerts stimulatory effect on the growth on breast cancer cells in the low concentration range [52]. The anti-cancer activity of FTY720 could be ascribed to the induction of protein phosphatase 2A (PP2A) activity and inhibition or degradation of sphingosine kinase 1, which is not dependent on FTY720 phosphorylation [53,54].

FTY720 has also been shown to downregulate S1PR1 in hepatocellular carcinoma [55]. In addition, FTY720 inhibited the SphK1/S1P/S1PR1 axis and abrogated NF-κB/IL-6/STAT3 signaling, which reduced the severity of colitis and colitis-associated cancer [40]. Up-regulation of S1PR1 and STAT3 expression was shown to correlate with poor survival of colorectal cancer patients, which additionally advocates targeting S1PR1 to attenuate malicious feed-forward amplification loop involving S1PR1/STAT3 signaling as a strategy to combat colon cancer [56]. The activation of S1PR1/STAT3 amplification loop seems to be an inherent feature of other cancer types as well [57,58] and targeting this signaling axis using S1PR1 antagonist FTY720 has already yielded promising results. For example, co-treatment with gemcitabine and FTY720 inhibited S1PR1-STAT3 activation loop and suppressed cell migration and invasion in pancreatic carcinoma cell line and SCID mice model for pancreatic cancer [49].

Several studies in colon cancer have also corroborated the ability of FTY720 to potentiate anticancer efficacy of chemotherapy drugs and to reverse chemoresistance. For example, co-administration of FTY720 with either 5-fluorouracil, SN-38 or oxaliplatin exerted an additive anti-proliferative effect in colorectal cancer cells [59]. One of the molecular mechanisms underlying anti-cancer effects of FTY720 in colorectal cancer cells included restoration of the activity of tumor suppressor protein phosphatase 2A (PP2A) and the resulting suppression of the activity of its targets AKT and ERK1/2 [59]. Another study demonstrated the ability of sub-toxic concentrations of FTY720 to significantly increase the response of multidrug-resistant colon cancer cells to doxorubicin and etoposide by inhibiting the activity of P-gp and MRP1, two ABC proteins known to confer cancer drug resistance [60]. FTY720 was also shown to restore sensitivity to anti-EGFR monoclonal antibody cetuximab in cetuximab-resistant colorectal cancer models both in vitro and in vivo accompanied by inhibition of tumor growth, induction of apoptosis and enhanced mice survival [61]. At the molecular level, the re-sensitization to cetuximab in resistant models induced by co-administration with FTY720 was associated with the inhibition of the of EGFR, AKT and MAPK activities, suppression of SphK1 activity and reduced S1P levels. Altogether, these studies clearly demonstrate that FTY720 could be used as an adjunct to standard chemotherapy to increase therapeutic response and improve survival in colorectal cancer patients.

## 4. S1P Production and Degradation in Colon Cancer

### 4.1. Sphingosine Kinases 1 and 2

Sphingosine kinase 1 (SphK1) is increased in many types of human cancers and has been recognized as a contributing factor in carcinogenesis, chemoresistance and poor patient outcome. SphK1 promotes V12Ras-dependent transformation and the growth and survival of cancer cells while inhibiting apoptosis and conferring resistance to γ-irradiation and chemotherapeutic agents [62]. No mutations linked with cancer have been identified in SphK1. However, cancer cells demonstrate a reliance on this enzyme for their growth and survival, the so-called a non-oncogenic addiction. High expression of SphK1 correlates with CRC progression, aggressiveness, distant metastasis and poor overall survival of the patients suffering from colorectal carcinoma [57,63,64]. Higher expression of SphK1 in cancer tissues in comparison to normal surrounding colonic mucosa was confirmed by immunohistochemistry analysis of CRC patient tissue samples [63,65]. Importance of SphK1 in the regulation of colon carcinogenesis was also shown in mice models with SphK1 knockout that exerted lower susceptibility to colon cancer development induced by azoxymethane (AOM, colon carcinogen) in comparison with wild-type mice, whereas SphK1 overexpression in intestinal epithelial cells enhanced AOM-induced colon tumor [63]. Different in vitro studies have also adduced a wealth of evidence to support oncogenic role of SphK1 in colon cancer cells. Growth-inhibitory effects elicited by SphK1 inhibition in colon cancer cells depend upon p53 functional activity since reduction of colon cancer cell growth and induction of apoptosis induced by SphK1 inhibition by specific inhibitor SK1-I is more enhanced in wild-type p53 cells than in cells with inactivated or mutated p53 [66]. SphK1 inhibitor SK1-I induced transcriptional activity of p53 protein ensued by rise in the levels of its pro-apoptotic downstream target genes leading to the induction of intrinsic apoptotic cell death, which points to an inverse correlation between p53 and SphK1 activities. However, down-regulation of SphK1 activity could also trigger modes of cell death other than apoptosis. Thus, inhibition of SphK1 activity by SK1-I in colon cancer cells also promotes autophagy in p53-dependent manner [66]. Furthermore, down-regulation of SphK1 activity by specific inhibitor PF-543 (Figure 3) led to induction of programmed necrosis in colorectal carcinoma cell lines HCT116, HT29 and DLD-1, primary cells derived from CRC patients and HCT-116 xenograft in SCID mice, which resulted in improved survival outcome in mice [67]. This study demonstrated that PF-543 had potent in vitro and in vivo anti-tumor activity independent of SphK1 initial expression, which completely results from a regulated induction of necrosis and not of apoptosis [67].

SphK1 coupled to ERK1/2 signaling is able to regulate autophagy via mTOR downregulation and consequent up-regulation of the ULK1 (Unc-51 like autophagy activating kinase) activity, as shown in HT29 colorectal carcinoma cell line. Although autophagy can be considered either pro- or anti-tumor mechanism, in colorectal cancer cells it is thought to be a marker for aggressive phenotype and adaptive module for CRC cancer cells as a response to stress stimuli such as chemotherapy. Utilizing potent autophagy inhibitors is considered possible modality for chemosensitization of CRC patients to standard therapy [68].

SphK1 acts in concert with different molecular mediators to promote colon cancer cell growth and survival. One of them includes COX-2 whose higher expression at protein and mRNA level was detected in colorectal carcinoma tissues [69,70,71,72]. The indication that COX-2 is under the control of SphK1 activity in colon cancer comes from the study showing that down-regulation of SphK1 in HT-29 human colon cancer cells reduces the expression of COX-2 and diminishes the generation of its enzymatic product prostaglandin E2 (PGE2), whereas over-expression of SphK1 in normal intestinal epithelial cells increases COX-2 expression [73]. The same study also showed that S1P induced COX-2 expression and PGE2 production in HT-29 cells, which suggests that SphK1/S1P axis promotes colon carcinogenesis at least partially by upregulating COX-2 expression and the resulting PGE2 production. COX-2 positively regulates the expression of matrix metalloproteinases (MMPs), enzymes catalyzing degradation of extracellular matrix which facilitates cancer invasion and metastasis. In colon cancer cells, up-regulation of matrix metalloproteinase-2 (MMP2) mRNA levels is associated with induction of COX-2 expression, whereas down-regulation of COX-2 by specific inhibitors leads to a decrease in MMP2 and MMP9 activity [72,74,75]. Additional studies in colon cancer have demonstrated the ability of SphK1 to also induce the expression of matrix metalloproteinases 2/9 [57,76], and given the correlation between SphK1 activity and COX-2 expression, it seems that SphK1 and COX-2 converge on matrix metalloproteinases to promote colon cancer growth and progression. Induced expression of matrix metalloproteinases 2/9 (MMP-2/9) and concomitant activation of FAK/AKT signaling pathway resulting from over-expression of SphK1 facilitates epithelial-mesenchymal transition (EMT) and increases migration capacity of human colon cancer cells HT29, while suppression of SphK1 reduces EMT and migratory potential and decreases the expression of p-FAK, p-AKT and MMP2/9 [57]. This study indicates that SphK1 contributes to metastatic progression of colon cancer by triggering EMT via up-regulation of MMP2/9 expression accompanied by activation of FAK/AKT signaling pathways [57]. Indeed, the role of SphK1 as a positive regulator of EMT in colon cancer was corroborated by other studies as well [77]. Other mechanisms by which SphK1 induces the production and secretion of MMP-2/9 in colon cancer cells to promote cell proliferation and invasiveness include the activation of ERK1/2 and suppression of p38 MAPK pathways [76].

Immunohistochemistry and Western blot analyses of colon cancer tissues and normal colonic tissues showed higher expression of SphK1, FAK (focal-adhesion kinase) and p-FAK in cancerous tissues, which was in correlation with histological grade, stage and metastasis. Focal adhesions are structures that enable adhesion of the cells to the extracellular matrix and regulate cell attachment, spreading and migration. The dynamics of the turnover of focal adhesions determines the rate of cancer cell migration. The involvement of S1P metabolism in the regulation of focal adhesions formation and dynamics in colorectal cancer was demonstrated in the study revealing that treatment of primary colorectal cancer cells with FTY720 diminished the number of focal adhesions [78]. Importantly, down-regulation of both S1P-producing enzymes SphK1 and SphK2 by siRNA reduced the formation of focal adhesions in primary colorectal cancer cells, which was accompanied by suppression of FAK activity. SphK-dependent regulation of FAK activity was also shown by Xu et al. [77] who reported that treatment of colorectal cancer cells with SphK1 inhibitor SKI-II abolished FAK activity to suppress EMT and colon cancer cell migration. Similarly, Liu et al. reported on the correlation between the expression of SphK1 and FAK or p-FAK, and showed increased expression of activated FAK as well as some other cell adhesion molecules including intercellular cell-adhesion molecule-1 (ICAM-1) and vascular cell adhesion molecule-1 (VCAM-1) in colon cancer LOVO cells induced by SphK1 over-expression, while the opposite effect was observed with the suppression of SphK1. ICAM-1 and VCAM-1 are considered the key players in SphK1/FAK-mediated carcinogenesis, metastasis and increased cell motility, and their higher serum levels may serve as prognostic markers for tumor progression and metastasis in patients suffering from colorectal cancer [79].

Given that SphK1 plays a central role in the activation of signaling pathways responsible for increased epithelial-mesenchymal transition, which is an important factor contributing to the development of chemoresistance [80,81], the mechanisms by which SphK1 modulates treatment response and development of chemoresistance in colon cancer have been the subject of investigation in different studies. For example, Kawahara et al. [82] explored the correlation between Sphk1 activity and the expression of CD44 as a possible mechanism underlying the resistance to oxaliplatin in CRC cell lines. CD44, cell surface glycoprotein, is known mediator of signaling pathways modulating the response of cancer cells to cytotoxic stimuli [83]. In addition to enabling cell survival, CD44 has been confirmed as an initiator of tumorigenesis and molecular marker for cancer stem cells [83,84] and has been linked with cell invasion and metastasis of colorectal carcinoma cells [85]. Increased expression levels of SphK1 and CD44 were observed in oxaliplatin-resistant colon cancer cells in comparison with parental cells [82]. Positive regulation of CD44 expression in ERK-dependent manner was detected in response to both, SphK1 overexpression and exogenous S1P stimulus in oxaliplatin-sensitive colon cancer cells. The suppression of CD44 expression was induced by inhibition of either SphK1 activity or by blocking S1PR2 receptor leading to down-regulation of CD44 expression levels and ERK activity in resistant colon cancer cells. Findings from this study suggest that oxaliplatin resistance in colon cancer cells involves the activation of SphK1/S1P/S1PR2 signaling axis that activates ERK signaling pathway resulting in the induction of CD44 expression [82]. However, activated ERK could also induce the activation of SphK1 to produce S1P creating thus ERK1/2/SphK1 positive feedback loop that drives the expression of CD44 and maintains the resistance to oxaliplatin. The importance of sphingosine kinase-mediated signaling in oxaliplatin resistance in colon cancer was also confirmed by Nemoto et al. [86] who demonstrated that colon cancer RKO cells with high activity and expression of SphK1 but also SphK2 exerted poor sensitivity to treatment with oxaliplatin in comparison with HCT116 colon cancer cells with low activities of both SphK isoforms that were sensitive to oxaliplatin. Expectedly, inhibition of both isoforms of sphingosine kinases by either pharmacological inhibitor or using siRNAs increased cytotoxic effects of oxaliplatin in the resistant RKO cells by inducing increase in the levels of pro-apoptotic C16-ceramide as a result of its increased production by ceramide synthase and sustaining the activation of pro-survival AKT signaling. In addition to oxaliplatin, SphK1 also mediates the effects of 5-fluorouracil (5-FU) in colon cancer, since SphK1 knockdown or pharmacological inhibition enhanced sensitivity of colon cancer cells to 5-FU [64]. Targeting SphK1 has also shown promising results in increasing the effectiveness of targeted therapies in colon cancer. Down-regulation of SphK1 activity by pharmacological inhibitor N,N-dimethylsphingosine (DMS) (Figure 3) or siRNA restored sensitivity to cetuximab in colorectal cancer cells with intrinsic or acquired resistance to cetuximab, whereas over-expression of SphK1 precluded cetuximab-induced apoptosis [61]. The role of SphK1 in resistance mechanisms to cetuximab was additionally corroborated by the finding that SphK1 expression significantly correlates with response to cetuximab in colorectal cancer patients.

Unlike SphK1, less is known about the mechanisms by which SphK2 regulates the development of CRC. In general, there is a controversy surrounding the role of SphK2 in cancer pathogenesis and treatment response with some studies showing pro-apoptotic and anti-tumorigenic functions of SphK2 and others indicating a pro-survival and oncogenic role. In colon cancer, pharmacological inhibition of SphK2 by ABC294640 (Figure 3) inhibits growth and induces apoptosis in vitro by suppressing the activity of pro-survival AKT-mTOR signaling pathway and activating pro-apoptotic JNK pathway, and inhibits the growth of HT-29 colon cancer xenografts in nude mice [87], which indicates oncogenic role of SphK2 in colorectal tumorigenesis. Indeed, significantly higher expression of SphK2 at protein and mRNA levels was reported in tumor tissues from primary CRC patients in comparison to matched normal mucosa and was associated with lymph node and distant metastasis [88]. Knockdown of SphK2 expression by siRNA curtailed proliferative capability of colon cancer LoVo cells and inhibited their migratory and invasion potential suggesting that SphK2 confers malignant phenotype of colon cancer cells. The same study [88] found a decline in MYC levels upon down-regulation of SphK2 expression, which suggests that growth-promoting role of SphK2 in colon cancer is associated with MYC, the latter being an important mediator of cell differentiation, survival, metabolism and chemoresistance with a demonstrated role in CRC tumorigenesis [89].

Some evidence has surfaced to suggest that the function of SphK2 is determined by its cellular levels with low-level SphK2 overexpression promoting cell proliferation and survival, and high-level overexpression of SphK2 decreasing cell proliferation and inducing cell death [90]. Low-level overexpression of SphK2 results in the activation of pro-survival and anti-apoptotic signaling pathways including AKT and ERK1/2, whereas high-level SphK2 has an opposite effect. The finding that low-level of SphK2 overexpression is able to induce neoplastic transformation in vitro and promote tumorigenesis in vivo sheds a new light on the role of SphK2 in cancer pathogenesis [90].

The regulatory mechanisms that control SphK2 expression have been poorly investigated. Study in colon cancer cells has indicated that specific stress stimuli such as serum deprivation increases mRNA, protein and enzyme activity of SphK2 but not SphK1 [91]. Transcription factor CREB, whose activation was preceded by JNK activation, was identified as the major inducer of SphK2 transcription under serum deprivation conditions in colon cancer cells. While serum deprivation conditions enabled a continuous albeit slower proliferation of colon cancer cells, glucose depletion promoted apoptosis and hypoxia inhibited cell proliferation in colon cancer cells [91]. The effects of glucose deprivation and hypoxia in colon cancer cells could be associated with a specific regulation of SphK2 expression which was attenuated under these stress conditions. Thus, SphK2 seems to play different roles in colon cancer cells under different stress conditions.

Recent study has provided evidence that fatty acid synthase (FASN), a key enzyme of de novo lipid synthesis, upregulates sphingolipid metabolism to promote metastatic phenotype in colorectal cancer [78] which brings into close association de novo lipogenesis and sphingolipid metabolism in the regulation of colon cancer progression. FASN over-expression in PDX model and SW480 colon cancer cells led to an upsurge in the expression of SphK2 but also of SphK1 by activating ERK1/2, while pharmacological inhibition of FASN specifically suppressed the expression of SphK2 in primary colorectal cancer cells and PDX model of metastatic colorectal cancer. In addition, a significant correlation between the expression of FASN and SphK2 at mRNA and protein level was detected in primary colorectal cancer. These findings posit that FASN positively regulates sphingolipid metabolism through the expression of sphingosine kinases, in particular Sphk2, and that targeting FASN/SphK/S1P signaling could provide novel therapeutic strategy for treating metastatic colorectal cancer [78].

Pharmacological inhibition of SphK2 activity has already proven as a promising chemosensitization strategy to increase therapeutic response in cancer. Preclinical studies have adduced a wealth of evidence to support the ability of specific SphK2 inhibitor ABC294640, which is currently being tested in cancer patients in phase I/II clinical trials, to increase cancer cell response to chemotherapy. For example, treatment with ABC294640 increased sensitivity of endocrine therapy-resistant MDA-MB-231 (highly aggressive, invasive and poorly differentiated triple-negative breast cancer) cells and chemoresistant MCF-7TN-R cells to doxorubicin and etoposide mediated by inhibition of NF-κB signaling [92]. In addition, enhanced cytotoxic effects of gemcitabine in human pancreatic cancer cells [93] and increased anti-myeloma effects of Bcl-2 inhibitor (ABT-199) [94] were achieved in combination with ABC294640. Study in colon cancer also revealed that low concentration of ABC294640 significantly improved growth-inhibitory and cytotoxic effects of 5-FU and cisplatin in colon cancer cells in vitro [87]. Similarly, knockdown of SphK2 enhances apoptosis-inducing effects of doxorubicin, which could be ascribed to down-regulation of p21 independently of p53 [95]. SphK2 positively modulates p21 expression in order to block doxorubicin-induced apoptosis by inducing cell cycle arrest and preventing cell death [95]. An overexpression of p21 can contribute to inhibition of apoptosis in human colorectal cell line HCT116 in p53-dependent or independent manner, thus indicating that the control of the p21 expression can contribute to sensitization to anti-cancer agents [96]. Given the ability of SphK2 to regulate p21 levels, targeting SphK2 to reduce the expression of p21 should be further explored as a potential strategy for augmenting the anticancer therapeutic efficacy of chemotherapy drugs.

Besides standard therapy, a vast number of natural compounds have been found to exert anti-proliferative and pro-apoptotic effects in colorectal cancer and have been investigated as possible agents for combined treatment with standard therapy to improve treatment outcome in colorectal cancer [97]. For some of them, SphK2 and its product S1P have emerged as key mediators of therapeutic response and resistance in colon cancer cells, as comprehensively described below.

Luteolin, 3′,4′,5,7-tetrahydroxyflavone (Figure 3), is a natural flavonoid contained in different types of plants including fruits, vegetables, and medicinal herbs with demonstrated anti-proliferative and anti-metastatic properties in colorectal cancer [98,99,100,101]. One of the mechanisms behind its growth-inhibitory effects in colon cancer cells includes induction of apoptosis due to luteolin-induced inhibition of ceramide transport from the ER to the Golgi apparatus, which decreases ceramide metabolism to complex sphingolipids and results in the accumulation of pro-apoptotic ceramide [102]. Cytotoxic effects of luteolin could be attributed to reduced intracellular levels of S1P resulting specifically from luteolin-induced inhibition of SphK2, which leads to suppression of intracellular S1P-stimulated activation of AKT independently of S1P receptors. As a consequence of inhibition of AKT activity, the ER-Golgi transport of ceramide is blocked and sphingolipid balance impaired resulting in the promotion of apoptotic cell death.

Sodium butyrate (NaBT), a naturally occurring short-chain fatty acid (Figure 3) produced as a by-product of carbohydrate metabolism, has arisen as a possible chemopreventive agent for colorectal cancer because of its demonstrated anti-migratory, anti-proliferative and pro-apoptotic effects in colorectal cancer cells [103,104]. The effects of NaBT in colon cancer cells are mediated by SphK2 which acts as a negative regulator of NaBT-induced apoptosis, as evidenced by the study showing that down-regulation of SphK2 by siRNA potentiates NaBT-induced apoptosis in HCT116 colon cancer cells, whereas its overexpression diminishes apoptosis rate [105]. Further mechanistic study into NaBT-induced apoptosis revealed the key role of protein kinase D (PKD) in this process. PKD is a serine/threonine kinase belonging to the Ca^2+^/Calmodulin-dependent kinase superfamily, which regulates cell proliferation, survival, migration, angiogenesis and protein/membrane trafficking [105]. Treatment of colon cancer cells with NaBT activates PKD that induces the activity of SphK2 and stimulates its translocation from the nucleus into the cytosol where SphK2 accumulates and inhibits NaBT-induced apoptosis. Indeed, PKD knockdown using siRNA resulted in a decrease in NaBT-induced Sphk2 phosphorylation and increased incidence of apoptosis in HCT116 colorectal cell line [105]. Additional clues to the mechanisms by which PKD regulates SphK2 activity and cellular transport in colon cancer cells in response to treatment with NaBT were provided by the study showing that NaBT elicited early ERK1/2 activation, and down-regulation of ERK1/2 activity in HCT116 colon cancer cells by specific UO126 inhibitor reduced SphK2 activity and its translocation from the nucleus [106]. The similar effect was accomplished by down-regulation of SphK2 using specific siRNA in combination with UO126 inhibitor, both resulting in decreased translocation of SphK2 from the nucleus to cytoplasm ending up with the sensitization effect to NaBT-induced apoptosis. Importantly, this study found that induced ERK1/2 was able to enhance PKD-mediated SphK2 activation and its consequent translocation to the cytoplasm to further negatively regulate NaBT-induced apoptosis in colon cancer cells, and provided an evidence to demonstrate that activation of ERK1/2 is an upstream event that precedes the induction of PKD and SphK2 in response to NaBT [106].

All-trans retinoic acid (ATRA), (Figure 3) metabolite of vitamin A, has been studied as anti-cancer agent due to its potent effect in regulating cell growth, proliferation, angiogenesis and cell death [107,108]. ATRA’s main mode of action includes interaction with retinoic acid receptors (RARs), among which RARβ is the most abundant in human body. Two independent studies [109,110] showed that HT-29 colon cancer cell response to ATRA was minimal despite a significant elevation in the RARβ expression. Interestingly, it seems that what lay behind the unresponsiveness to ATRA was high expression of SphK2, since the response of colon cancer cells to ATRA was enhanced when SphK2 was knocked down by siRNA. In addition, S1P antagonized the effects of ATRA on colon cancer cell proliferation, ATRA-stimulated RARβ expression, G1 phase arrest and apoptosis induction, and these events were reversed by down-regulation of SphK2. Findings from these studies indicate that S1P/SphK2 may play a protective role against cytotoxic effects of ATRA in colon cancer cells and encourage further studies into targeting this signaling axis to improve therapeutic efficacy of retinoids in colon cancer treatment.

### 4.2. S1P Lyase

Decreased catabolism of S1P has been observed in different cancer types where the loss of S1P lyase activity was shown to promote chemoresistance and to drive neoplastic transformation and tumorigenesis [111]. For example, the study in patients with prostate cancer demonstrated that S1P lyase expression and activity was significantly down regulated in tumor tissues in comparison with normal adjacent tissues and was correlated with the aggressiveness of prostate cancer [111]. Reduced degradation of S1P in prostate cancer was paralleled by increased enzymatic activity of SphK1 in cancer tissues, which suggests that S1P metabolism in prostate cancer is shifted towards the S1P production. Silencing of S1P lyase activity in prostate cancer cells gave rise to their improved survival after irradiation and chemotherapy drug docetaxel, whereas its overexpression increased the sensitivity of prostate cancer cells to irradiation and chemotherapy as a result of altered ceramide/S1P balance in favor of ceramide production [111]. This and similar studies [112] suggest that up-regulation of S1P lyase activity could represent an efficient strategy to counteract chemoresistance.

Several lines of evidence confirm an aberrant regulation of S1P degradation in colon cancer as well, although with great controversy. Thus, Uranbileg et al. [44] detected increased expressions of S1P lyase and S1P phosphatase 1 at the mRNA level in colon cancer tissues in comparison with adjacent non-tumor tissues [44]. Oncogenic role of S1P lyase in colon cancer was corroborated in vitro by demonstrating that S1P lyase silencing decreased proliferation and invasion of colon cancer cells, whereas its overexpression had an opposite effect [44]. On the contrary, Oskouian et al. [113] reported that the expressions of S1P lyase and S1P phosphatase 1 and 2 at the gene level were markedly reduced in human colon cancer tissues in comparison with adjacent normal tissues. Immunohistochemistry analysis confirmed decreased expression of S1P lyase in colon cancer tissues in comparison with the patient-matched normal colonic crypts. Another layer of evidence to support the tumor suppressor role of S1P lyase in colon cancer was provided in the same study by showing that the expression and activity of S1P lyase was decreased in adenomatous lesions of the mouse model of intestinal tumorigenesis [113]. Congruent with reports in other cancer types, loss of S1P lyase activity may be central to intestinal carcinogenesis.

There is ample evidence that S1P degradation is an important molecular event linking inflammation and colon cancer. In particular, intestinal S1P lyase has been shown to play protective role in colitis-associated cancer (CAC) [114]. Specifically, loss of S1P lyase activity in intestinal epithelium increases susceptibility to chemically induced colitis-associated cancer as demonstrated by increased crypt cell proliferation and increased formation of colon tumors in mice lacking intestinal S1P lyase subjected to chemically induced colitis in comparison with control mice with induced colitis [114]. Loss of S1P lyase activity in the gut epithelium facilitated early inflammatory responses in murine gut mucosa associated with STAT3 activation, STAT3-mediated target gene induction and induction of STAT3-activated miRNAs. Inhibition of STAT3 in mice lacking S1P lyase activity with chemically induced colitis-associated cancer resulted in the reduction of inflammatory response and decreased incidence of colon tumors, which indicates that pro-inflammatory and pro-tumorigenic effects of gut S1P lyase disruption are mediated by STAT3 activation [114]. Importantly, in mice with chemically induced CAC, oral administration of plant-type sphingolipids called sphingadienes increased the expression levels of S1P lyase in colon tissues, lowered the levels of activated STAT3, suppressed inflammatory response and reduced tumor formation. Findings from this study clearly indicate that restoration of S1P expression inhibits pro-inflammatory and pro-tumorigenic signaling in CAC and put forward S1P lyase as a target for chemoprevention in CAC.

Recent findings by Schwiebs et al. [115] indicate that compartment specific S1P lyase (*SGPL1*) knockout is critical for disease severity in CAC. Thus, *SGPL1* depletion in colon tissue cell compartment and intracellular S1P signaling result in fast growth of epithelial-driven tumors that facilitate specific immune microenvironment enabling further tumor growth. Lack of S1P lyase activity in tissue cells augments cancer-induced inflammation. Oppositely, *SGPL1* knockout in immune cell compartment leads to massive immune cell infiltration within colon tissue resulting in tissue damage and pathological crypt remodeling that induced delayed tumor formation [115]. This process is facilitated by extracellular S1P signaling derived from immune cells. Thus, SGPL deficiency in immune cells promotes inflammation-induced cancer. Findings from this study clearly demonstrate that the molecular mechanisms involved in inflammation-induced cancer versus cancer-induced inflammation involve different steps depending on the initiating cellular S1P source [115].

## 5. Conclusions

**Figure 4:**
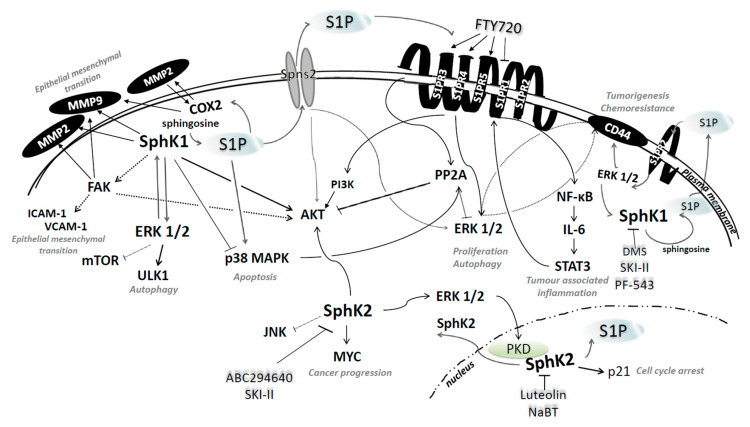
Sphingosine kinase/S1P signaling in the regulation of colon cancer cell behavior and fate and the corresponding pharmacological inhibitors. Findings from the available literature reveal a crosstalk between S1P metabolism and cellular networks that regulate colon cancer cell growth and survival. Sphingosine kinase 1 (SphK1) regulates the expression and activity of important players in colon cancer cell survival and metastatic progression. In response to various growth factors, activated ERK1/2 induces SphK1 activity to further stimulate pro-survival signals via activation of AKT and promote EMT through the activation of FAK and COX2 that converge on MMP2/9. SphK1-generated S1P can stimulate either receptor-dependent or independent signaling. S1P transported to the extracellular space by specific transporter proteins binds to and activates G protein-coupled receptors (S1PR1-3) in the plasma membrane to promote cancer cell survival and inflammation via downstream signaling resulting in the activation of PI3K/AKT, ERK1/2 and NF-κB/IL-6/STAT3. SphK1/S1P axis also interacts with CD44/ERK1/2 signaling through S1PR2 receptor. Sphingosine kinase 2 (SphK2)- produced S1P in the nucleus binds to histone deacetylases (HDAC1 and 2), inhibits their activity and induces *p21* gene transcription. Increased ERK1/2 activation could also potentiate protein kinase D (PKD) activity to facilitate activation and transport of SphK2 to the cytoplasm, where it can promote cancer progression via interaction with MYC and inhibition of pro-apoptotic JNK signaling.

Studies into the role and functions of S1P and the enzymes regulating its metabolism in colon cancer have provided a novel perspective on the intricate interplay between sphingolipid signaling and cellular signaling networks and have shed new light on the processes central to cancer progression and metastasis including cell growth, proliferation, migration, invasion and inflammation (Figure 4). Investigation into how modulation of S1P metabolism shapes colon cancer cell fate and response to treatment is propelled by the advancements in the design and synthesis of novel compounds specifically targeting sphingolipid metabolism. Such efforts have already yielded several drug candidates whose therapeutic efficacy is currently being clinically tested in cancer patients. Identification of novel molecular targets that act in concert with oncogenic sphingolipid mediators to protect cancer cells from cytotoxic insults would open the door to new combination treatments to overcome drug resistance in colon cancer.
